# Communicative patterns and social networks between scientists and technicians in a culture of care: discussing morality across a hierarchy of occupational spaces

**DOI:** 10.1080/14649365.2021.1901976

**Published:** 2021-04-06

**Authors:** Nathalie Nuyts, Carrie Friese

**Affiliations:** Sociology Department, London School of Economics and Political Science, London, UK

**Keywords:** cultura del cuidado, animales de laboratorio, redes sociales, moralidad, soins, care, recherche sur les animaux, tuer/mort, études en sciences et technologies, géographie animale, Culture of care, laboratory animals, social networks, morality

## Abstract

Communication between scientists and animal technicians is considered important for creating a ‘culture of care’ in facilities that use animals in scientific research. For example, the Brown report, which investigated alleged failures of animal care at Imperial College London, noted the physical and social separation between animal technicians and scientists as a problem that delimited a culture of care. This paper seeks to better understand the communicative relationships between scientists and animal technicians in this context. We conducted a survey of scientists working in the UK who use animals in their research (n = 230), asking who they spoke with about various aspects related to using animals in research. We found that scientists communicated with technicians about operational issues, while they spoke with other scientists about experimental design as well as moral questions and concerns. We probe the meaning of these communicative relationships using narrative analysis of semi-structured, qualitative interviews conducted with consenting survey respondents (n = 14). Analytically, this paper seeks to bridge social network analysis with geographies of care through a shared concern with relations of power.

## RESUMEN

La comunicación entre científicos y técnicos en animales se considera importante para crear una ‘cultura del cuidado’ en las instalaciones que utilizan animales en la investigación científica. Por ejemplo, el informe Brown, que investigó supuestos fallos en el cuidado de los animales en el Imperial College de Londres, señaló la separación física y social entre técnicos animales y científicos como un problema que delimitaba una cultura del cuidado. Este artículo busca comprender mejor las relaciones comunicativas entre científicos y técnicos en animales en este contexto. Realizamos una encuesta a científicos que trabajan en el Reino Unido y que utilizan animales en su investigación (n = 230), y les preguntamos con quién hablaron sobre varios aspectos relacionados con el uso de animales en la investigación. Descubrimos que los científicos se comunicaban con los técnicos sobre cuestiones operativas, mientras hablaban con otros científicos sobre el diseño experimental, así como sobre cuestiones e inquietudes morales. Investigamos el significado de estas relaciones comunicativas mediante el análisis narrativo de entrevistas cualitativas semiestructuradas realizadas con encuestados que dieron su consentimiento (n = 14). Analíticamente, este artículo busca unir el análisis de las redes sociales con las geografías del cuidado a través de una preocupación compartida por las relaciones de poder.

## RÉSUMÉ

Les sciences de l’animal de laboratoire représentent un aspect complexe et sujet à controverses dans les relations entre les humains et les animaux, parce que sa pratique implique la souffrance ou la mise à mort d’animaux, délibérément ou accidentellement. Par conséquent, la recherche sur les animaux a fait l’objet de fortes préoccupations et réglementations sur le plan éthique dans le Royaume-Uni, afin de réduire les épreuves et les douleurs subies par ces bêtes dont les corps vivants modèlent entre autres les maladies humaines. Cette communication s’appuie sur des recherches ethnographiques longitudinales et des entrevues détaillées entreprises avec des techniciens animaliers de laboratoire débutants dans des universités du Royaume-Uni entre 2013 et 2015, ainsi que des perspectives venant d’entretiens avec des acteurs principaux du bien-être animal. Dans notre analyse, nous étudions quatre dimensions essentielles des soins dans la recherche animale de laboratoire. (i) les compétences et les affinités spécifiques qui sont nécessaires ; (ii) l’importante d’expériences préalables en soins animaliers (iii) l’influence des cadres institutionnel et affectif et (iv) l’expérience nécessaire pour mettre fin à des vies animales. Nous suggérons que des notions de soins différentes se déroulent en parallèle, pas seulement les seuils de douleur autorisés à être infligés aux animaux de recherche suivant les protocoles de recherche, mais aussi la douleur des techniciens animaliers dans les processus de soins et de mise à mort des animaux. Pour conclure, nous nous prononçons en faveur d’une meilleure concertation dans la coexistence des soins et des souffrances à travers les débats au sein de la géographie sur les soins, le care et les relations entre les humains et les animaux.

In 2013 the animal rights organization British Union for the Abolition of Vivisection (BUAV) published ‘Licensed to Kill’, a video created by an undercover BUAV member who gained employment at an animal facility at Imperial College London (ICL). The video presented explicit images of mice being decapitated with a guillotine as well as mice waking up from anaesthesia during experimental procedures. The video led to allegations of incompetence and neglect on the part of scientists at ICL and sent shock waves through both the British public and scientific community. The Home Office immediately responded with an official investigation (Home Office, 2014), while ICL requested an independent investigation (Brown, 2013). The government’s Animals in Science Committee (ASC) (Animals in Science, 2014) wrote a report and made recommendations based upon these two investigations.

Across these three reports, several unsatisfactory practices were described. For example, a significant proportion of the scientists were found to be unaware of the responsibilities attached to their licences from the Home Office, which are required to conduct animal experiments. Many scientists did not keep adequate records, did not comply with humane endpoints and did not report to the Home Office when these humane endpoints were exceeded. There was an unwillingness to adopt the principle of the 3Rs (replace, reduce and refine animals in research) in practice, as is required by law. There was insufficient staffing of the ICL animal facility, and communication between scientists and animal facility staff was deemed substandard. The ‘culture of care’ was labelled poor by the Home Office report. The Brown Report (Brown, 2013, p. 24) in part located this in the organizational hierarchy that puts research staff in a position of power vis-à-vis animal care staff, stating: ‘The existence of a “them and us” view (taken of each other by both animal care staff and research colleagues) also did not appear to contribute to the recognition of the expertise of the animal care staff. Nor would this improve the confidence of animal care staff so that they felt able to challenge research staff on animal care and welfare issues.’

## The problem

The desire and need to create a ‘culture of care’ in facilities that use animals as part of scientific research is a relatively new discourse for enacting an organizational morality. While the idea of creating a culture of care in animal facilities was circulating before the case at Imperial, there is a discursive link between the Imperial case and the discourse of a ‘culture of care’ in Britain. One of the lessons of the Imperial case has been that there are negative consequences to the hierarchical relationship between scientists and technicians, which alienates scientists from the lives of the animals used in their research while delimiting the power of technicians to care for the animals – and as a result for the organization. Yet there are diverse ideas and practices regarding how to go about creating a culture of care. While it is generally agreed that a culture of care is about going above and beyond regulated minimal standards to provide a good environment for both animal technicians and laboratory animals alike (Greenhough & Roe, 2018; Nc3rs, 2019; Norecopa, 2019), how to do this remains an open question.

Facilitating good communication patterns between scientists and technicians is generally viewed as a crucial part of creating a culture of care, but Friese has found through participant observation research that the nature of this communication can range from distanced communication through email that ensures a strict division of labour to making more proximate working relationships between technicians and scientists such that their work is purposefully blurred. And so while empowering technicians is a focus within the culture of care discourse (Greenhough & Roe, 2018), there are ongoing questions regarding how best to organize the relationships between two different occupational groups and two different spaces in order to create a ‘culture of care’. This paper probes how scientists understand and enact communicative relationships as part of using animals in their research. In the process, and more analytically, we seek to bridge social network analysis with geographies of the space and ‘scope’ of care (Atkinson et al., 2011).

## Analysing communication in the spaces of science and care

Social science scholarship on laboratory animals has emphasized the importance of understanding how scientists think about and respond to the welfare of animals used in their experiments (Davies, 2012a; Hobson-West, 2012; Sharp, 2019), and to the debates over their use in research (Birke et al., 2007). Rob Kirk (2008, 2010, 2012, 2014), Tone Druglitrø(2018) and Greenhough and Roe (2011, 2018) have explored the professionalization of laboratory animal science in this context. They build upon and extend earlier ethnographic research that explored the relationships between animal technicians and scientists and the corresponding division of labour (Arluke, 1991; Birke et al., 2007; Michael & Birke, 1994). Where scientists are socialized to distance themselves from the animals and to see animals as tools, animal technicians cannot engage in this kind of emotional distancing (Birke et al., 2007, p. 98; Sharp, 2019). A concern with animal care has been thought of as something that distinguishes technicians from scientists. Love and care for animals has been a crucial part of creating a science of nurturing for laboratory animals (Druglitrø, 2018; Friese, 2019; Kirk, 2010, 2014), while also raising questions and concerns about the burden of this emotional labour in the context of work-related stress and burnout (Greenhough and Roe this issue; Friese et al., 2019). This paper contributes to this literature by considering how scientists engage with technicians in a relationship that has historically been hierarchical.

Geographies of care help us to understand the significance of the different spaces that scientists and animal technicians work in, which is linked to issues of power through hierarchy. Geographers have used spaces of care to understand the relationalities and subjectivities that emerge through not only utilitarian needs but also (and potentially more importantly) an interestedness in another (e.g. Conradson, 2003). Here the proximate relations of care in space have been emphasized, such as those between the technician and the animal. Meanwhile, Bowlby and McKie (2019) have emphasized the spatial aspects of care in their ecological approach that links experiences with environments of care. Milligan and Wiles (2010) show the importance of networks in the landscapes of care to these environments, which link various actors and actants across spaces. Geographies of care thus provide ways to think across proximate and distant caring (see also Little this issue; Gorman & Davies, 2020).

These ecological perspectives are crucial for both understanding and doing a ‘culture of care’ with laboratory animals, as there are two very different environments (animal facility and laboratory) that relate and are related to differently, depending upon the type of research being conducted. For example, the scientist conducting an *in vivo* experiment will likely spend a great deal of time within the animal facility – and so with the research animal and animal technicians – while the scientist conducting an *ex vivo* experiment likely will not. This paper seeks to understand if and how relationships between scientists and animal technicians are enacted through communication (or not) across different environments and types of experiments, and what issues bring these two different occupational groups together (or not).

We focus here specifically on communication patterns across professional groups who occupy different spaces, extending the focus on discourse and interpretive repertoires in the existing scholarship that has explored the content of communication on laboratory animals across different social groups. Holmberg and Ideland (2009) have, for example, explored how laboratory workers and members of animal ethics committees talk about transgenic animals in ways that render transgenic mice both ordinary and important. They emphasize how the shared interpretive repertoires used across different social groups means that human agency is unilaterally silenced, making the topic of not making and using transgenic mice unspeakable. Dam et al. (2020) have further analysed how ‘zones of unknowing’ make animals invisible in discussions of human biomedicine. Both focus on the discursive side of communication. By contrast, in this paper, we explore who scientists speak with about what kinds of issues in order to understand how those in a position of power enact communicative networks (or don’t) on animals in bioscience. Rather than emphasize the interpretive repertoires or discourses used in those conversations, we focus on the networks created through communication itself.

Milligan and Wiles (2010, p. 737) suggest that care is ‘a complex network of actants and actions with multidirectional flows of activity and connections’; we seek to operationalize these flows through communication itself. As Little emphasizes (this issue), ideas about people shape the capacity to care within and across environments. It was the capacity for technicians to care that was dangerously delimited in the Imperial case, in part because of scientists’ ideas about technicians. This paper explores the communicative patterns of scientists in order to further probe the structural dynamics at play when creating a culture of care in laboratory science that use animals. In the process, we seek to bridge social network analysis as a theory-methods package with the network thinking that informs ecological approaches to geographies of care. Both seek to ‘capture wider social values and power relations’ (Atkinson et al., 2011, p. 564) that emplace care socially.

Geographies of laboratory animals have almost exclusively used qualitative research methods to date, creating a rich scholarship on embodied care. There have been calls to extend the methods used in studying laboratory animals specifically – and animals in society more generally – in order to address more widespread social processes that condition animals in society (Johnson, 2015). This paper addresses this call by using quantitative research methods and redirecting analytic attention to communicative practices. Through the metaphor of networks, we aim to connect qualitative research on embodied care in geography with quantitative research on communicative practices in social network analysis. As a culture of care is increasingly being understood as a tool for regulators, and as something that can be measured (e.g. the culture of care barometer in the NHS), it is important to offer critical analyses of these metrics through metrics themselves.

## Materials and methods

To further probe the communicative relationships between scientists and technicians, two data sets were combined for analysis: 1) a survey that we designed and implemented, which explored scientists’ (n = 230) attitudes regarding animal care as well as how often and with whom scientists spoke with others about animal care and welfare; and 2) follow up, qualitative interviews that we conducted with consenting survey respondents (n = 14) that asked, among other things, how scientists narrated the salience of animal care for their research and how they believed this had changed over time. These data sets represent the first work package in an ongoing study funded by the Wellcome Trust. The research project as a whole asks how much and why scientists in the UK think that animal care is important to scientific knowledge production, how this value is practiced and where this idea comes from. This question was a response to earlier social science scholarship on laboratory animals, which positioned animal care as something that animal technicians did and that was separate from scientists’ work (Birke et al., 2007; Lynch, 1989). The other two work packages include ethnographic research (Friese, 2019; Friese & Latimer, 2019) and historical research (Holmes & Friese, 2020).

Based on the survey and follow-up qualitative interviews with scientists, we have elsewhere reported on patterns in scientists’ attitudes regarding the importance of animal care for scientific knowledge production (Friese et al., 2019). Here, we focus on patterns in how scientists reported on their communication practices.

### Survey sample

For the survey, we created a database of UK-based scientists who had published an article on biomedical research that used animals between 1 January 2011 and 31 December 2014. This database included 49,164 unique authors, from which a random sample of 2000 scientists was taken. Accounting for possible outfall due to the mobility of researchers and missing contact information, our goal was to have a final sample of about 1000 scientists. We contacted our final sample of 1164 scientists in the last week of June 2015 with the request to participate in our research by completing an online survey. To optimize the response rate, the initial email was followed up with two e-mail reminders and a paper version of the survey was distributed by post. The survey had a response rate of 37% though not all respondents completed the survey fully. In addition, due to the way the initial database was constructed, some of these respondents were not actively using animals in experimental research. We received 172 valid and complete surveys. Because scientists in industry and government were underrepresented due to the sampling method, this group was additionally targeted by snowball sampling, which resulted in an additional 58 valid and complete surveys. This paper is based upon an analysis of both the random and snowball sample, totalling 230 valid and completed surveys. A control variable for the samples is added in the analyses to examine possible effect differences between the random and the snowball sample.

We surveyed scientists because care work has historically been relegated to animal technicians, and has not been seen as part of ‘science’. Further, animal technicians typically do not appear as authors on the journal articles that we used to create the sample, which speaks to the entrenched hierarchies involved in scientific knowledge production that we seek to further understand. This means that we do not have corresponding data regarding patterns in how animal technicians’ report on their communication practices. Our sampling method means that the analysis is one-sided and thus limited; future research using this same survey technique but on a population of technicians would be beneficial to provide a point of comparison.

### Survey measures

Opinions regarding animal care and welfare were assessed in multiple ways within the survey. First, respondents were asked to indicate the importance of animal care on a scale of 1 (Not at all important) to 5 (Extremely important). The importance was judged with regard to twelve topics (see Table 1 for an overview). Second, respondents were asked to indicate on a scale from 0 (Not at all responsible) to 10 (Fully responsible) how responsible they are for the care of animals in their research. Third, communicative behaviour was assessed using a sociometric procedure where respondents were asked to complete a roster. The roster was accompanied by the instructions: ‘Think about all the people at work with whom you have spoken about animal care and welfare in scientific research over the last month. We would like to know a little more about each of these people. Please complete a line in the table below for each individual (maximum 10 people).’ In the first column, we asked how the person relates to the respondent. Seven possible answers were provided: Co-worker, Line manager, Member of your staff, Student, Technical or supportive staff from animal facility, Scientist from another lab/institution, or Other. In the second column, the respondent was asked to characterize the overall topic of conversation. Possible answers were: operational, experimental setup, moral/philosophical and regulatory. In contrast to the first column, in this column, multiple answers could be given.

**Table 1.** Distribution of responses (in percentages).

Q: How important is animal care for …	Not at all important	Somewhat important	Important	Very important	Extremely important	N
Regulatory compliance	0	2	4	23	71	223
Acquiring funding	1	7	19	36	37	213
Translating research to other species	2	5	12	34	47	217
Keep up-to-date with most recent trends in animal care	6	10	21	36	27	221
High quality data	0	4	5	28	63	222
Reproducing findings	0	2	7	26	65	222
Designing experiments	1	1	14	29	55	221
Publishing in scientific journals	2	14	20	33	31	219
Ethical/moral reasons	1	1	3	19	77	221
High quality science	0	4	8	29	60	221
Education and training of scientists	1	4	10	26	60	218
Public support for research	1	3	7	18	71	218

The percentages in each row do not total to 100 due to rounding.

We used the words ‘moral/philosophical issues’ as opposed to ‘ethical issues’ deliberately. In piloting the survey, the word ethical was elided with formal regulation as respondents questioned the difference between ethical issues and operational/technical issues. We used ‘moral/philosophical’ because we wanted to capture issues and concerns that scientists may encounter and experience that are not only or not necessarily addressed by codified ethics and regulations. As Lesley Sharp (2019) has shown, morality captures everyday and ordinary ethics that can be informal, serendipitous and creative. What this means for this paper is that ‘ethics’ per se was not explicitly addressed in this part of the survey, and so in some instances could have been positioned as part of the regulatory and in other instances positioned as part of the moral by those who answered the survey. We used qualitative interviews to further elaborate upon the meaning and significance of the moral.

Demographic variables were also addressed. The survey asked the respondents about their age, gender and nationality. For gender, respondents could tick one of the three boxes: woman, man or other. Since no respondent ticked the box ‘other’, gender is treated as a binary variable. Age and nationality were measured with an open-ended, write-in question. Respondents’ occupational position was also measured with an open-ended, write-in question and was recoded to the following categories: manager, PhD student, post-doc, faculty staff, tech support, senior management. Finally, respondents were asked how often they interact with animals (daily; several times per week; once a week; once every two weeks; once per month; less than once per month; never) and how many animals they use as part of their research in an average week (1–5; 6–10; 11–20; 21–30; more than 30). Both interaction with animals and number of animals used in research were recoded as dichotomized variables: interaction frequency with animals was recoded into 0 = ‘once a month or more often’ and 1 = ‘never’ and number of animals into 0 = ‘one or more’ and 1 = ‘none’. These dichotomized variables correlate weakly (r = .37, sig = .000) which means that there is only a weak relation between the frequency of interaction and the number of animals used.

### Sample description

The composition of the survey sample shows the diversity of respondents that participated in the survey. Forty-seven percent of our respondents were women while 53% were men. The average respondent is 42 years old (SE = 11). Sixty-nine percent of the respondents are British nationals. Seventy-three percent of the respondents work within academia, 15% within industry, 10% in research institutes, and the remaining 2% work in other institutions (i.e. charity or government). Respondents were asked to indicate the field(s) in which they worked from a list based on the Home Office licence application forms. All 26 fields from the list are present in our sample. The smallest field – dentistry – represents only 2% of the respondents, while the largest field – molecular biology – employs 30%. Other large fields in the sample are immunology/immunity (23%), physiology (21%), genetics (19%), and cancer research (19%). The species which scientists work with was assessed using the same lexicon as Home Office licence applications. Ninety-two percent of the respondents work with mammals, while 15% work with non-mammals. Seven percent of the respondents work with non-Home office regulated invertebrates. The mammals most often used by respondents in our sample are mice (73%), rats (37%), dogs (13%), pigs (8%), rabbits (7%), and monkeys (6%).

### Qualitative interviews

Survey respondents were asked if they were willing to be contacted regarding a follow-up qualitative interview, to which 59 agreed. All 59 respondents were contacted after the survey had closed. Fourteen have been interviewed. Nine have declined to be interviewed. Thirty-four have not responded. Two e-mail addresses were no longer in use. The qualitative interviews ask respondents to describe: 1) how they came to be interested in science; 2) the role of animal care in their work; and 3) how they define animal care, welfare, husbandry and a culture of care. Interviews took place in person when possible (n = 3) or by Skype (n = 11).^1^ All but one interview was digitally recorded; when recording was not permitted, we both took detailed notes during the interview. The recorded interviews were professionally transcribed and double-checked for accuracy. The interview recordings, notes and transcripts were given codes to ensure anonymity.

Initial interviews were open coded by us both independently, along the lines of constructivist grounded theory coding (Charmaz, 2014). These open codes were then consolidated into 29 codes, and the remaining interviews were coded accordingly. The codes were then read and re-read in conjunction with survey findings. The codes that we focus on here include: 1) care; 2) care protocols; 3) culture of care; 4) division of labour; and 5) shifting ideas of care.

## Findings from survey and interviews

The findings are presented in two sections. The first section examines how important scientists reported animal care to be. The second examines whom scientists spoke with about different aspects of animal care. By adopting a social networks approach we focus specifically on everyday relations that structure a culture of care. Work with laboratory animals is a joint task involving scientists, veterinarians and technical staff; communication amongst these groups has been highlighted as crucial for developing a ‘culture of care.’ Using social networks to explore communicative patterns allows us to focus on specific types of social resources that are deemed relevant to quality animal care (such as being able to talk about moral issues, find technical support, receive operational information, etc.) among the different individuals and occupational groups. In this way, it complements how a culture of care is measured through tools like the NHS barometer, which more generally looks at whether the staff feel valued, respected and supported by colleagues, the team leader and the Trust.

### Animal care – values

Both the survey and the interview data give insight into the value scientists attach to animal care and welfare. In the survey, respondents were asked to indicate the importance of animal care for varying aspects of scientific work (see Table 1) using a scale of 1 (Not at all important) to 5 (Extremely important). In general, the responses are skewed toward the extremely important end of the scale, with high percentages responding 4 (Very important) and 5 (Extremely important). More than 90% of the respondents reported that animal care is very or extremely important for ‘regulatory compliance’, ‘producing high quality data’, ‘reproducing findings’ and ‘ethical/moral reasons’. While less so, the responses remained skewed towards very or extremely important for ‘acquiring funding’, ‘keep up-to-date’ and ‘publishing’. Further, the respondents in our sample reported feeling responsible for the care of animals in their research. On a scale from 0 to 10 – from ‘not at all responsible’ to ‘fully responsible’- the average respondent scored 7.05 (SD = 3.05). Around 30% of our respondents indicated they are fully responsible, which is in line with Home Office definitions of project licence holders specifically.

These findings indicate that the respondents attach high levels of importance to animal care for scientific, regulatory and ethical reasons as well as for gaining public support. This was confirmed through conversations with scientists in follow-up qualitative interviews. Feelings of personal responsibility and attributing a high value to animal care were common themes throughout the interviews. Care served a utilitarian value for the scientists we interviewed. Animals are a special type of scientific material, as living sentient beings. To serve their purpose, these animals require at a minimum clean housing, fresh water and food to survive. For their bodies to serve as high-quality scientific materials, these animals require adequate housing to be comfortable and healthy but also companions, quality handling and enrichment to avoid stress. However, the sense of responsibility that scientists expressed in the interviews also went beyond this type of utilitarian value. Animal care was also understood as an obligation both to the wider society and to the animals. Terms like ‘responsibility’ and ‘honour’ were often used to articulate this. We see this idea of care explicitly expressed through the trope of a ‘culture of care’ as follows:

I can’t obviously speak for everyone, but certainly most of the people I work with, they genuinely care. There’s a new culture of care for animals. Because I think people now realise that it is a life that you’ve got to take care of. And that it’s not just part of your experiment; it’s not just a reagent that you can throw away. There’s a lot more to it. And I think the concept is definitely starting to take over the whole of the industry.

INTERVIEWEE 7, Skype (9 June 2016)

This scientist describes the ‘culture of care’ as ‘taking hold’ where new ideas about laboratory animals become genuinely felt, thus exceeding a utilitarian understanding of care.

Further, care for animals as part of science was frequently discussed as a shift or a change. This theme was expressed as follows:

When I did my PhD in early 1980s - and that was before the 1986 Act was written in - and I think people’s attitudes and the systems and processes that we had in place at that time were not as heavily regulated as they are now. And I think people’s attitudes were different. And I saw some poor animal health during that time. … I suppose especially from the emotional point of view, you don’t like to see animals with poor care and suffering as a consequence of that. But it also made me realize that it makes bad science.

INTERVIEWEE 9, Skype (13 June 2016)

While the scientists we interviewed did not condemn the behaviour of others, they would condemn the practices associated with an earlier time period. Not caring about animal wellbeing and allowing animals to suffer particularly through stress were considered commonplace in the past. And these practices are today considered both ethically and scientifically negligent and unacceptable. Further, these practices are described as bad science. Other interviewees would comment on how poor care created confounding variables, and thus legitimated care as a crucial part of science according to the logic of objectivity. The high value placed on animal care in this project’s ethnographic research work package and in the wider literature (Dam & Svendsen, 2018; Davies, 2012a; Davies et al., 2018; Nelson, 2018; Svendsen & Koch, 2013) was thus paralleled by the survey findings and qualitative interviews.

### Animal care – communicative behaviour

We now ask if and how the overwhelming importance that scientists attach to animal care is reflected in their behaviour. For the purposes of the survey, we assessed behaviour through communication. This allows for a different approach to studying values and practices when compared to the ethnographic work package. In the survey, the respondents were asked to give information (i.e. occupational role, topic of discussion) about the individuals with whom they had spoken about animal care and welfare in scientific research over the last month. Sixteen per cent said that they had not discussed the topic with anyone. The majority (48% of the respondents) had discussed the topic with one to three other people. A smaller group (18%) had discussed the topic with four to seven individuals. And eight per cent had discussed animal care and welfare with ten or more individuals in the past month. Ten per cent of the responses for this variable are missing.

We built a logistic regression model to better understand why individuals did not communicate with others about animal welfare in the last month. Here the presence or absence of relations was the dependent variable and individual and job level was the independent variables (Table 2). We found that people who are not currently using any animals in their research are also not conversing with others about animal welfare and care, which is what we would anticipate. Individual-level factors (age, gender, nationality) do not have a significant effect. However, not directly interacting with animals in the research that is currently being conducted does not significantly relate to discussing animal welfare issues with others. *In other words, the fact that a scientist works with tissues derived from the animal after its death – and therefore has less interaction with the living animal than, for example, in* in vivo *scientific research – is not associated with whether or not they discuss animal care*. This finding was surprising to us in the context of this project’s ethnographic research work package and the wider literature (e.g. Birke et al., 2007).

**Table 2.** Logistic regression of individuals and job characteristics on the absence or presence of communication relations (N = 182).

	Relations absent or present		Exp(B)
Constant	1.74	*	
	(0.81)		
Age	0.02		
	(0.02)		
Gender^a^	−0.65		
	(0.43)		
Nationality^b^	−0.68		
	(0.48)		
Number of animals: none^c^	−1.43	+	0.24
	(0.84)		
Interaction frequency animals: never^d^	−0.27		
	(0.68)		

^a^Reference category is woman

^b^Reference category is non-UK

^c^Reference category is one or more

^d^Reference category is once a month or more frequently

*p < .05, + p < .10

**Figure 1.**
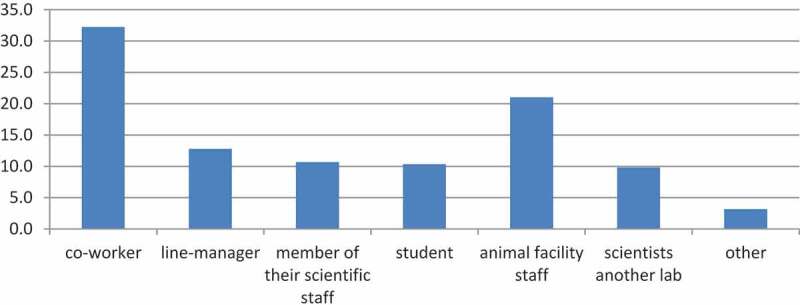
Percentage of relations (N = 574) according to alter category.

The following analysis of the communicative networks is based only on those respondents who have discussed the topic of animal care and welfare with others. The scientists were asked to label their relationship with their alters (i.e. the individuals to whom one is directly connected) along the following categories: 1) a co-worker, 2) line-manager, 3) member of their scientific staff with the respondent thus being their manager, 4) student working in the research lab, 5) technical or support staff from the animal facility, 6) scientist from another lab or institution, or 7) other. Care and welfare issues were most often discussed with co-workers and animal facility staff, which comprised 53% of the total relations (see Figure 1). The other categories of alters (line-managers, members of their scientific staff, students and scientists from other labs) each comprise around 10% of the total number of relationships.

That said, 29% of the respondents did not have any discussions with co-workers and 47% had not had any conversations with animal facility staff. Furthermore, 60% of the respondents did not report any communication relations with line managers, 77% did not report any communication relations with members of their scientific staff, 70% did not report any communication relations with students and 73% did not report any communication relations with scientists from other labs.

For each of the relationships, the respondents could indicate which of the four possible topics (operational, experimental design, moral/philosophical and regulatory issues) animal care and welfare was discussed with each individual. We found that 84% of the respondents discussed experimental design and 75% discussed operational issues with one or more person. A smaller group of respondents (55% and 58%, respectively) discussed moral or regulatory issues. Thirty per cent of the respondents had discussed all four topics in the previous month. In 51% of the relationships, only one topic is discussed. Only 8% of the relations discussed all four topics.

A final quantitative analysis examines whether respondents’ characteristics and the characteristics of the person with whom they are speaking can explain the content of the conversation. This allowed us to ask: who is (not) involved in the discussions about operational, experimental design, moral/philosophical and regulatory issues that arise in caring for laboratory animals.^2^ Four multilevel logistic regression models were built that take into account the nested structure of our data (relations embedded within individuals).^3^ Table 3 shows the results of these four models. The dependent variables are for the presence (or absence) of a relationship discussing animal care and welfare in terms of an operational issues (model 1), an experimental design issue (model 2), a moral/philosophical issue (model 3), and finally a regulatory issue (model 4). The independent variables are the same for the four models: four respondents’ characteristics and one alters’ characteristic. The respondents’ characteristics are age, gender, nationality and occupational position. The alters’ characteristic inserted in the model is the alters’ occupational position in relation to the respondent as also presented before in Figure 1.

**Table 3.** Results of multilevel logistic regression models.

	Model 1 Operational		Model 2 Experimental		Model 3 Moral		Model 4 Regulatory	
Constant	0.35		−0.91		−3.08	*	−4.25	***
	(0.83)		(1.09)		(1.28)		(1.28)	
Age	0.02		0.00		−0.04		0.02	
	(0.02)		(0.02)		(0.02)		(0.02)	
Gender^a^	0.17		0.45		0.57		1.23	***
	(0.24)		(0.33)		(0.38)		(0.37)	
Nationality2	0.19		−0.16		0.55		−0.55	
	(0.26)		(0.36)		(0.40)		(0.40)	
Position^c^: manager	0.26		−0.18		1.37		1.35	
	(0.49)		(0.66)		(0.75)		(0.76)	
Position^c^: PhD student	0.27		−0.60		0.86		0.83	
	(0.54)		(0.72)		(0.82)		(0.86)	
Position^c^: post-doc	0.03		0.47		1.27		1.12	
	(0.45)		(0.61)		(0.69)		(0.71)	
Position^c^: faculty staff	−0.30		−0.43		0.48		0.38	
	(0.48)		(0.64)		(0.75)		(0.75)	
Position^c^: technical support	−0.27		−1.39	*	0.81		0.00	
	(0.52)		(0.71)		(0.81)		(0.84)	
Position^c^: senior management	0.06		0.34		4.10	**	2.59	
	(0.83)		(1.10)		(1.25)		(1.42)	
Alter^d^: co-worker	−1.68	***	1.23	***	0.92	*	0.11	
	(0.30)		(0.31)		(0.39)		(0.33)	
Alter^d^: line-manager	−1.39	***	1.09	**	0.15		0.34	
	(0.37)		(0.39)		(0.49)		(0.42)	
Alter^d^: member of scientific staff	−1.01	*	1.7	***	1.49	**	0.57	
	(0.40)		(0.45)		(0.54)		(0.47)	
Alter^d^: student	−1.34	***	1.08	*	2.42	***	0.35	
	(0.38)		(0.43)		(0.49)		(0.45)	
Alter^d^: scientists another lab	−1.62	***	1.27	**	1.03	*	0.07	
	(0.39)		(0.44)		(0.50)		(0.45)	
Alter^d^: other	−1.46	*	1.22		2.26	**	1.95	*
	(0.64)		(0.76)		(0.77)		(0.93)	
Snowball sample	−0.04		0.08		0.99	*	0.64	
	(0.31)		(0.43)		(0.48)		(0.49)	
Random Part								
$\sigma_{u}^{2}$	0.39		1.45		1.64		1.94	
	(0.19)		(0.37)		(0.45)		(0.47)	

^a^Reference category is woman

^b^Reference category is non-UK

^c^Reference category is non-academic scientists

^d^Reference category is animal facility staff

*** p < .001, ** p < .01, *p < .05

Note. All models have 517 units on level 1 (i.e. relationships) and 149 units on level 2 (i.e. individuals).

The first model examines the effects of the independent variables on whether or not operational issues involving animals in science are discussed. Significant effects are found for the alter categories. The reference category for this variable is the animal facility staff. For each of the categories of alters in the model (i.e. co-workers, members of staff, student, scientists from another lab or line-managers) the results show a significant, negative correlation. The chances of discussing an operational issue with these alters (which are all scientists) are thus lower than discussing it with animal facility staff. In other words, *operational issues are more likely discussed with animal facility staff than with scientists.*

The second model addresses conversations regarding experimental design where we find significant positive correlations for the alter categories co-workers, members of staff, students, scientists from another lab and line-managers. The chance of discussing issues regarding animals and experimental design are higher when the ‘alter’ is a scientist than when the ‘alter’ is animal factility staff. *Questions and concerns regarding animals in experimental design are thus more likely discussed with scientists rather than with animal facility staff.*

The third model addresses discussions about moral/philosophical issues. Similar to experimental design, the chance of discussing a moral issue is higher when the ‘alter’ is a scientist (i.e. co-workers, members of staff, students, scientists from another lab) as opposed to animal facility staff. *Questions and concerns regarding morality in research involving animals are thus more likely discussed with scientists rather than with animal facility staff*. The effect for line-managers does not significantly differ from the animal facility staff, which is the reference group.

*The fourth model shows that there was no significant effect found in terms of who regulatory issues are discussed with.*

In conclusion, for the three first models (i.e. operational, experimental and moral conversations), occupational position of the ‘alter’ – or the individual with whom the survey respondent is reporting a communicative relationship with – correlates significantly with the dependent variables. The type of issue discussed by the survey respondent depends upon the occupational position of the ‘alter’ with whom they had the conversation. *The survey indicated that scientists tend to have conversations with animal facility staff regarding operational issues. However, they tend to discuss moral and experimental design issues with other scientists*. While we were not surprised that scientists would speak with technicians about operational issues and other scientists about experimental design, we were surprised that scientists did not speak with technicians about moral issues. The ethnographic research highlighted that technicians are generally considered guardians of the animals’ best interests, having the most everyday knowledge of the animals. It is thus surprising that this ‘intimate knowledge’ (Raffles, 2002) would not be sought out (see Friese, 2019).

In the interviews, the communicative patterns found in the survey data are confirmed. Here scientists responded to our survey findings as follows.

Well, these people, they know the details of the animals, they know more about what a mouse does over a 24-hour period than I do. So yes, we discuss with them quite a lot. Obviously, the experiment, at the end of the day, has to be an experiment well designed to answer the question we need to do, so it’s not … I’m not going to go out and say, please design my experiment for me; no, I can design my experiment, this is how it is best to do it, but then the fine-tuning, we talk with them, of course.

INTERVIEWEE 6, Skype (3 June 2016)

The only time we would ask about experimental issues would be if we were told, you can’t do that, and then the question is why and what that means. It’s a fair point. The animal technicians have the expertise and the care of the animal. I mean … they handle them every hour of every day in their work, so I think it would be beneficial to bring that in more, but we certainly don’t.

INTERVIEWEE 7, Skype (9 June 2016)

We see here the idea of a division of labour, such that scientists are responsible for designing experiments and animal facility staff are responsible for caring for animals. There is at times overlap between these two types of work, for example, when animal facility staff tell scientists that they cannot conduct their research in the manner planned. However, the scientists here articulate a strong division of labour that was also seen in the survey.

Only one of the scientists we interviewed articulated why scientists are more likely to discuss moral issues with other scientists rather than animal facility staff in a manner that did not rely on a division of labour. She stated:

I’ve always felt, in the grand scheme of things, that the NACWOs and the techs have moral high ground, and that they’re the good guys and we are not … . It doesn’t surprise me that people would talk to their peers rather than them, because … they’ve got the moral high ground … . It’s exposing talking to a NACWO. Not that I haven’t talked to NACWOs about personal stuff, but not about the morality of animals

INTERVIEWEE 14, Skype (25 November 2016)

Interestingly, this scientist does not locate the lack of communication between animal facility staff and scientists in a division of labour per se, but rather in her own struggle to accept the moral implications of her work. Lack of communication is here a means to avoid confronting uncomfortable aspects of scientific work by communicating with those who occupy a shared position.

Little emphasizes (this issue) that ideas about people shape the capacity to care within and across environments. We see here two different sets of ideas that scientists have about themselves and about animal technicians that shape their communicative practices and thus their capacity to care. On the one hand, a division of labour between science and care work is presented as a dominant discourse and reproduces a hierarchy wherein the scientist is dominant vis-à-vis the animal technician. However, another discourse also circulates wherein the morality of animal technicians reverses this hierarchy and puts the technician in a superior position. This reversal does not, however, facilitate communication either as it leads the scientist to avoid communication so as to avoid judgement.

## Discussion and conclusion

This paper has explored connmunicative patterns of scientists in order to further probe the meaning of a culture of care in laboratory science that use animals. A clear division of labour between scientists and technicians has been problematized in recent years through empirical research (Dam & Svendsen, 2018; Davies, 2010, 2012a, 2012b; Davies et al., 2018; Druglitrø, 2018; Kirk, 2010, 2014, 2016; Nelson, 2018; Sharp, 2019; Svendsen & Koch, 2013; Svendsen et al., 2018) The Imperial Case has made this descriptive problematization a prescriptive necessity, wherein a strict division of labour between technicians and scientists has been deemed a problem in and of itself. The move to develop a culture of care in laboratories has thus emphasized the importance of communication and collaboration between scientists and technicians, laboratories and animal facilities. In this context, this paper has explored the communicative practices of scientists.

Our survey measured communicative relationships and juxtaposed reports of those relationships onto ongoing questions regarding what a culture of care looks like in the life sciences. In this sense, the survey differs from other measures of a culture of care that focus on experiences of relationships. Tools like the NHS barometer look at whether staff feel valued, respected and supported by colleagues, the team leader and the Trust. This contrasts from our social network approach that explores the practices of relations. As a result, we found that scientists turn to technicians with operational issues and speak with fellow scientists about experimental design and moral concerns. The most surprising finding here was that scientists speak with other scientists – as opposed to animal technicians – about moral issues. These findings show the potential importance of operationalizing a culture of care as not only experientially felt but also relationally structured in its measurement.

The qualitative interviews help us to better understand the survey findings. Many scientists emphasized a division of labour to explain why they would speak with scientists about experimental design and technicians about technical issues. In the process, the reason why they would speak with scientists about moral issues became bound up in a division of labour, even if the technician is generally understood as the moral guardian of the animal. However, an alternative perspective was also articulated if only by one person: that (some) scientists do not want or feel able to discuss moral issues with animal technicians precisely because they are the moral guardian of the animal. In other words, scientists may not want to open themselves up to critique through such a conversation. Conversations can make people feel vulnerable, and there is a question of how that vulnerability can be addressed in creating a culture of a care.

This raises questions about how relations can be forged in the context of two different hierarchies that both seem to disable conversation, at least from the scientists’ perspective. Some research groups address this by integrating the communication networks of scientists and technicians in making their working relationships more proximate. For example, in the ethnographic research, Friese saw some organizations mandate that scientists carry out animal care work like cage cleaning that is normally done by the animal technician. But this is not the only solution, from either a social network or geographies of care perspective. For example, social network analysis has shown that there are often ‘brokers’ who facilitate and support communication between groups (Borgatti, 2006; Burt, 1992; Burt, 2005). Brokers are individuals who have the capacity to connect and transfer information between (physically, cognitively, or culturally) separated groups who do not necessarily trust each other. For example, the role of broker has been identified as crucial in the health care sector for setting up collaborative networks among different professional groups (Long, Cunningham and Braithwaite, 2013). Exploring who acts as brokers in the geography of laboratory animal care would be an important next step for the field.

It is important to note that communicative behaviours were not different when we compared scientists who work with tissues derived from the animal after its death to scientists who conduct experiments *in vivo* with a living animal. We had speculated that the materiality of the animal (parts) being used in the scientific experiment would be linked to ideas about animal care work and scientific research as well as communicative relationships. For example, Lynch’s (1989) ethnographic research in a neuroscience laboratory formed the basis for his now canonical distinction between the ‘naturalistic’ and ‘analytic’ animal. This distinction helps us understand much of genetic research that uses animal tissue after death, making it possible to separate animal care and science. We believed that distance from the living animal may result in fewer conversations about animal care. This interpretation was not reflected in the survey, however.

Conversely, ethnographic research focusing on *in vivo* experiments has shown how the naturalistic and analytic animals are entwined, such that animal care is a crucial part of producing a scientific fact (Dam & Svendsen, 2018; Friese, 2013; Nelson, 2018; Svendsen & Koch, 2013; Svendsen et al., 2018). We therefore speculated that *in vivo* scientists may have more communicative relationships and thus a greater social network around animal care. This might explain why some animal facilities insist that creating a culture of care requires dismantling a division of labour and creating working conditions that enable scientists and technicians to work in greater proximity. This interpretation was similarly not reflected in the survey findings.

Through a shared focus on networks, this paper has sought to bridge social network analysis with geographies of care. Both seek to ‘capture wider social values and power relations’ (Atkinson et al., 2011, p. 564) that emplace care socially. What social network analysis allows us to see is how general ideas that scientists have about themselves and about technicians shape their communicative practices, and this shapes if and how a culture of care takes shape within the organizations and institution of science. Social network analysis thus provides a way to operationalize through measurement the power relations that are of central concern to geographies of care.

## Notes

1. Interviewees were given the choice to have the interview take place by Skype or in person, in line with ethical requirements to conduct interviews at a time and a place that is convenient for the research participant.

2. We did not assess for the form of the conversation (e.g. in person, by email, by telephone).

3. The method used is proposed by Snijders, T., Spreen, M. and Zwaagstra, R. 1995 ‘The use of multilevel modeling for analysing personal networks: Networks of cocaine users in an urban area’, *Journal of quantitative anthropology* 5(2): 85–105. and de Miguel Luken and Tranmer de Miguel Luken, V. and Tranmer, M. 2010 ‘Personal support networks of immigrants to Spain: A multilevel analysis’, *Social Networks* 32(4): 253–62. The analysis is only executed on respondents with at least one reported relationship.
